# Radioprotective Effect of Melatonin in Reducing Oxidative Stress in Rat Lenses

**Published:** 2011-08-24

**Authors:** Alireza Shirazi, Gholam Hasan haddadi, Fahimeh Asadi-Amoli, Saeideh Sakhaee, Mahmoud Ghazi-Khansari, Abolghasem Avand

**Affiliations:** 1. Medical Physics Department, Faculty of Medicine, Tehran University of Medical Sciences, Tehran, Iran; 2. Medical Physics Department, Faculty of Medicine, Fasa University of Medical Sciences, Fasa, Iran; 3. Histology Department, Faculty of Medicine, Tehran University of Medical Sciences, Tehran, Iran; 4. Pharmacology Department, Faculty of Medicine, Tehran University of Medical Sciences, Tehran, Iran; 5. English Department, Faculty of Medicine, Fasa University of Medical Sciences, Fasa, Iran

**Keywords:** Free Radicals, Radiation, Melatonin, Radioprotector, Cataract

## Abstract

**Objective:**

Ocular morbidity is widely observed when radiotherapy includes the orbit. Oxidative stress generated by irradiation is responsible for this complication. In different studies, it has been shown that melatonin has antioxidative properties and a radioprotective role. The aim of this study was to evaluate the antioxidant role of melatonin against radiation-induced oxidative injury in rats' lenses after total cranial irradiation.

**Materials and Methods:**

Thirty-six adult female Sprague-Dawley rats were divided into six groups. Group I was the control group, group II only received total cranial gamma irradiation of 5 Gy, group III was exposed as the second group but at the dose of 8 Gy, group IV received 30 mg/kg melatonin 30 minutes prior to radiation plus total cranial irradiation of 5 Gy plus 5 mg/kg melatonin daily through intraperitoneal injection for ten days after irradiation, group V was treated similar to the fourth group, i.e. received irradiation plus melatonin, but at the dose of 8 Gy, and group VI only received melatonin (30 mg/kg on the first day and 5 mg/kg on the following days). Ten days after irradiation, all rats were sacrificed and their eyes were enucleated to measure the biochemical parameters i.e. malondialdehyde (MDA) and glutathione (GSH).

**Results:**

The levels of MDA in rat lenses increased and the levels of glutathione in lenses decreased after gamma ray irradiation but these parameters were still within normal limits in rats that received melatonin.

**Conclusion:**

It could be concluded that melatonin is useful in preventing radiation-induced oxidative injury due to its antioxidative and free radical scavenging properties.

## introduction

Eye morbidity is widely observed in patients receiving total-body irradiation (TBI) prior to bone marrow transplantation or radiotherapy for ocular or head and neck cancers (1-7). In some studies, it has been recognized that damage to DNA, proteins and lipids is responsible for eye morbidity and gamma radiation can induce these damages through free radical production (8). It is noteworthy that free radicals are naturally produced by some systems within the body, like ocular tissues, and under normal conditions, the antioxidant defense system within the body can easily handle free radicals that are produced. Gamma-ray exposure, however, causes an imbalance between free radical production and the antioxidant defense, which results in oxidative stress conditions. These conditions cause cellular damage and both early and late effects.

Investigations have shown that different antioxidants, such as melatonin, protect various tissues from the damaging effect of gamma-ray exposure (9, 10). Melatonin, which is the main secretory product of the brain pineal gland, is produced in many other tissues including the retina and lens (11). Antioxidant effects of melatonin are exerted through both direct and indirect mechanisms. Melatonin acts directly as a free radical scavenger, whereas the indirect actions of melatonin occur when it stimulates antioxidant enzymes, thus improving the endogenous antioxidant defense capacity of the organism (11, 12). The aim of this study was to investigate the possible radioprotective role of melatonin in rats' lenses against gamma radiation-induced oxidative injury after total cranial irradiation.

## Materials and Methods

### Chemicals

Melatonin (N-acetyl-5-methyoxytrptamin) was obtained from Sigma-Aldrich. It was dissolved in a minimal volume of ethanol (8mg/ml) and diluted with saline. All other reagents were obtained from Sigma (St. Louis, MO) and Merck (Germany) pharmaceutical companies.

### Animals

The experiment was performed on 36 adult female Sprague-Dawley rats that were 8-12 weeks old and weighed 180 g to 200 g at the time of radiation. They were fed with a standard rodent chow diet and water, and were kept in a windowless laboratory room with automatic temperature (22℃) and lighting controls (12 hours of light/12 hours of darkness). All procedures in this study were in accordance with the guidelines for care and use of laboratory animals adopted by the Ethics Committee of the School of Medicine at Tehran University of Medical Sciences.

### Experimental design

The rats were divided into six groups, each consisting of six animals. The first group served as the control group. The second group only received total cranial gamma irradiation of 5 Gy. Similar to the second group, the third group was exposed to total cranial gamma radiation but at the dose of 8 Gy. Group IV received 30 mg/kg melatonin 30 minutes prior to radiation plus total cranial irradiation of 5 Gy plus 5 mg/kg melatonin daily through intraperitoneal injection for ten days after irradiation. Group V was treated similar to the fourth group, i.e. received irradiation plus melatonin, but at the dose of 8 Gy, and group VI only received melatonin (30 mg/kg on the first day and 5 mg/kg on the following days).

### Irradiations

The rats were anesthetized with an i.p. injection of ketamin (60 mg/kg) and xylazin (20 mg/kg). Then, the rats were placed in the prone position. The rats in groups 2, 3, 4 and 5 were treated with Cobalt- 60 gamma irradiation (Theratron 760-C) with an output of 1.8 Gy/min and a source-to-skin distance of 80 cm. To increase lens dose to maximum, we threw a 2 mm thick damp towel over the eyes of the rats. Sham irradiation was performed on rats in groups I and VI and they were anesthetized but not irradiated.

### Sample preparation

Ten days after irradiation, the rats were anesthetized with ketamin and sacrificed by intra-cardiac KCl injection. Their lenses were then removed by a posterior approach. Later, they were washed, weighed, frozen and kept at -70℃.

### Biochemical survey

The rats' lenses were homogenized in 1 ml of 0.9% cold saline. Then, 0.2 ml of 25% trichloroacetic acid (TCA) was added to the homogenate and centrifuged at 5000 rpm for 15 minutes. The clear upper supernatant was used for measuring glutathione (GSH) content and the sediment was used for measuring the malondialdehyde (MDA) level. The MDA level was determined according to the thiobarbituric acid (TBA) method (13). Briefly, 2.5 ml of 0.05 M sulfuric acid and 3 ml of 0.2% solution thiobarbituric acid (TBA) were added to the sediment. The mixture was heated at 100℃ for 30 minutes in a boiling water bath. 4 cc of n-butanol was added to the cooled mixture and the sample was shaken vigorously. After centrifugation at 3500 rpm for ten minutes, the organic layer was taken and its absorbance read at 532 nm. MDA concentration was calculated from the standard curve and tetraethoxypropane (TEP) was used as standard for setting up the calibration curve.

Glutathione content was determined according to the method of Kuo and Hook (1982) (13). Briefly, 0.5 cc distilled water, 2 cc of 0.3 M disodium phosphate (Na_2_HPO_4_) and 0.5 cc of 0.04% 5, 5'-dithiobis- 2-nitrobenzoic acid (DTNB) were added to 0.5 cc of the supernatant and incubated for ten minutes at room temperature. The absorbance of the resulting yellow color was read against the blank at 412 nm and the GSH concentration was calculated from the standard curve. Pure GSH was used as standard for establishing the calibration curve.

### Statistical analyses

The biochemical data results were analyzed using oneway ANOVA and the t-test. Statistical analysis was performed using SPSS software (Statistical Package for the Social Sciences for Windows; version 16).

## Results

### Tissue MDA levels

**Fig 1:**
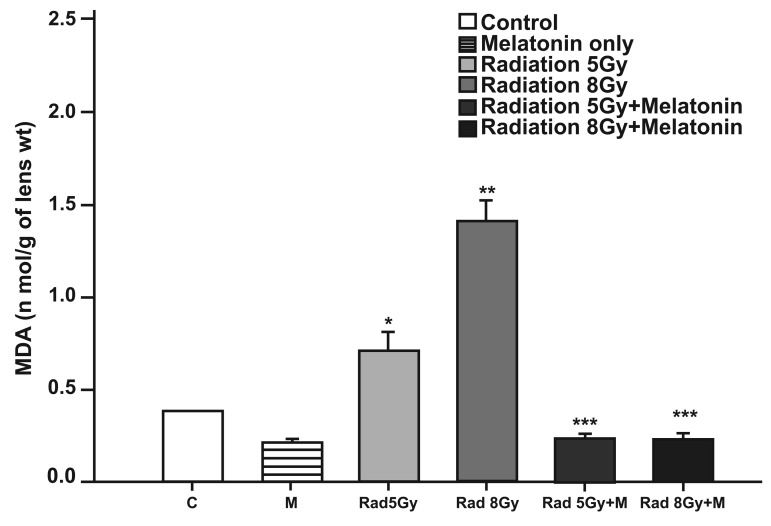
The effect of melatonin on the level of MDA in rats after total cranial gamma irradiation. Data represent mean standard error of mean. (n=6 animals per group). *p<0.05 (compared to control group), **p<0.002 (compared to the control group), ***p<0.001 (compared to the radiated groups).

Ten days after irradiation, the levels of MDA in lens tissues were found to be significantly higher in irradiation groups when compared to the control group (p<0.05 for 5 Gy irradiation compared to the control group and p<0.002 for 8 Gy irradiation compared to the control group), while melatonin treatment significantly reversed MDA levels back to control levels (Fig 1).

### GSH activity

**Fig 2:**
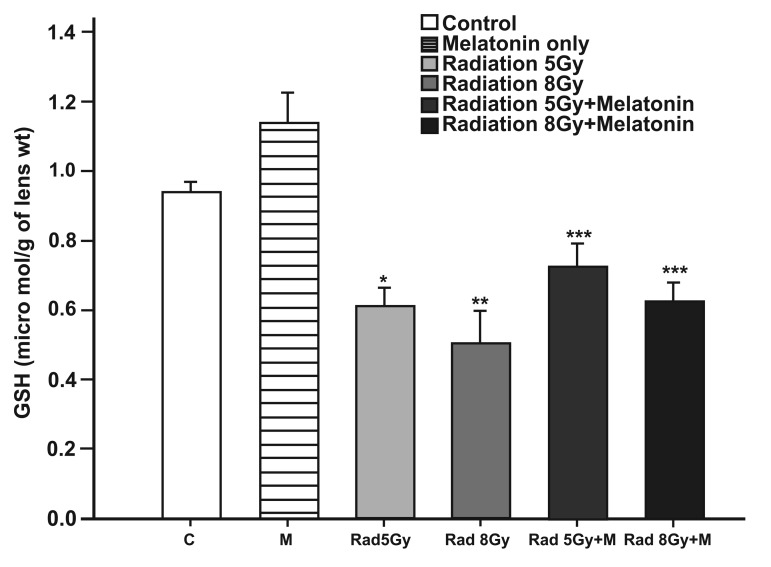
The effect of melatonin on GSH levels in rats after total cranial gamma irradiation. Data represent mean standard error of mean. (n=6 animals per group). *p<0.05 (compared to control group), **p<0.001 (compared to control group), ***p<0.05 (compared to radiated groups). Although significant differences were seen between irradiated and melatonin-treated groups in GSH concentration, we could not observe the biological effect of this antioxidant agent and concluded that these changes were not of clinical importance.

The levels of GSH in lens tissues significantly decreased after irradiation when compared to the control group (p<0.05 for 5 Gy irradiation compared to the control group and p<0.001 for 8 Gy irradiation compared to the control group), while melatonin treatment significantly reversed GSH levels back to the control levels (Fig 2).

## Discussion

The interaction between ionizing radiation and biological molecules can lead to the generation of free radicals and reactive oxygen species (ROS). When these free radicals and ROS are accumulated in the body, they can cause damage to cellular macromolecules (i.e., DNA, nucleic acids, lipids, proteins, and carbohydrates). The extent of this damage depends on exposed and absorbed doses, the duration of exposure, interval after exposure, and sensitivity of the tissues to ionizing radiation (11, 12). The ocular lens is one of the most radiosensitive tissues (14) and the potential of the ionizing radiation to produce cataracts has been confirmed since Chalupecky's studies in 1897 (15).

Investigation has shown that the most important effects of ionizing radiation on ocular lenses are as follows: 1. Inducing DNA damage in lens epithelial cells (2). 2. Damage to cellular membranes (plasma, mitochondrial and endomembrane systems), which is initiated by a process known as lipid peroxidation (13). 3. A decrease in the antioxidant defenses of the lens (i.e antioxidant enzymes such as catalase, superoxide dismutase, glutathione peroxidase, and non-enzymatic antioxidants such as glutathione) (16). All these changes disturb the function of the lens cells, increase light scattering in the lens, and cause cataracts.

Melatonin is a mammalian hormone which is synthesized from serotonin mainly in the pineal gland, but some is also synthesized in the retina, eye lens, bone marrow and lymphocytes. Melatonin plays a significant role in the regulation of many physiological events. It also has been known as a very powerful antioxidant. Most of the studies that have been performed on the antioxidant and radioprotective properties of melatonin (12,15-18) have emphasized these properties. Several reasons were considered in choosing melatonin as a radioprotector in this study. First, melatonin exerts direct antioxidant effects via its free radical scavenging properties and/or by inhibiting their generation. Second, melatonin exerts indirect antioxidant effects by stimulating antioxidant enzymes and inhibiting the activities of pro-oxidative enzymes. Third, melatonin is highly lipophilic and quite hydrophilic unlike Vitamin C and glutathione, which are only active in the aqueous phase, and Vitamin E, which is only active in the lipid phase. Fourth, melatonin is distributed ubiquitously in organisms and, as far as is known, in all cellular compartments. Finally, melatonin quickly passes through all biological membranes (17-19).

Some studies have reported that irradiation increases MDA formation as an end product of lipid peroxidation (17-20). In the present study, when rats were exposed to total cranium irradiation of 5 Gy and 8 Gy in a single dose, the MDA level of the lens significantly increased in comparison to the control group, but the levels of MDA in the lens in the "irradiation plus melatonin" groups significantly decreased when compared to the "irradiation only" groups. Our results are in agreement with published literature. These results demonstrate that melatonin clearly decreases lipid peroxidation in the lens induced by total cranium irradiation and decreases the level of MDA, thus indicating that using melatonin before irradiation, as a radioprotector, is useful. The mechanism of the inhibition of lipid peroxidation by melatonin probably includes the direct scavenging of hydroxyl radicals and single oxygen, which are both capable of initiating lipid peroxidation.

Some studies have reported that irradiation decreases tissue GSH concentration (20,21). Glutathione, as an antioxidant, protects cells against ROS and oxidative damage by participating in the cellular defense system (20). In the present experiment, although significant differences were seen between irradiated and melatonin-treated groups in GSH concentration, we could not observe the biological effect of this antioxidant agent and concluded that these changes were not of clinical importance.

While some studies have concluded that single fraction irradiation of 5 Gy to the total cranium of adult rats significantly increases grade 2 cataract formation ten days after irradiation (15,19), our results did not yield the same conclusion. In this study, similar to the aforementioned studies, we used adult albino female Sprague-Dawley rats and examined them every two days after irradiation for any development of clinical signs of cataracts for a period of ten days by a slit-lamp, but no signs of cataracts were observed. It is noteworthy that ocular lenses are late-responding tissues to irradiation and in fact, the incidence of histopathological changes and radiation- induced cataracts needs more time.

## Conclusion

Data obtained from this study showed that radiation exposure decreased levels of GSH and increased levels of MDA in the lens, but these values were within normal limits when melatonin was administered. These results emphasize the antioxidative and free radical scavenging properties of melatonin and indicate that melatonin can decrease the formation of late side effects of radiation, such as cataracts, by decreasing oxidative stress conditions. Therefore, concomitant melatonin administration during radiotherapy may protect ocular lenses against radiation-induced oxidative injuries.
